# Peer review in team-based learning: influencing feedback literacy

**DOI:** 10.1186/s12909-021-02821-6

**Published:** 2021-08-12

**Authors:** Annette Burgess, Chris Roberts, Andrew Stuart Lane, Inam Haq, Tyler Clark, Eszter Kalman, Nicole Pappalardo, Jane Bleasel

**Affiliations:** 1 Faculty of Medicine and Health, Sydney Medical School, Education Office, The University of Sydney, Sydney, NSW 2006 Australia; 2 Faculty of Medicine and Health, The University of Sydney, Sydney, NSW 2006 Australia

**Keywords:** Peer review, Team-based learning, Feedback, Team dynamics

## Abstract

**Background:**

Peer review in Team-based learning (TBL) exists for three key reasons: to promote reflection on individual behaviours; provide opportunities to develop professional skills; and prevent ‘free riders’ who fail to contribute effectively to team discussions. A well-developed process that engages students is needed. However, evidence suggests it remains a difficult task to effectively incorporate into TBL. The purpose of this study was to assess medical students’ ability to provide written feedback to their peers in TBL, and to explore students’ perception of the process, using the conceptual framework of Biggs ‘3P model’.

**Methods:**

Year 2 students (*n* = 255) participated in peer review twice during 2019. We evaluated the quality of feedback using a theoretically derived rubric, and undertook a qualitative analysis of focus group data to seek explanations for feedback behaviors.

**Results:**

Students demonstrated reasonable ability to provide positive feedback, but were less prepared to identify areas for improvement. Their ability did not improve over time, and was influenced by the perceived task difficulty; social discomfort; and sense of responsibility in providing written feedback.

**Conclusions:**

To increase student engagement, we require a transparent process that incorporates verbal feedback and team discussion, with monitoring of outcomes by faculty and adequate training.

**Supplementary Information:**

The online version contains supplementary material available at 10.1186/s12909-021-02821-6.

## Background

Peer review in Team-based learning (TBL) exists for three key reasons: to promote critical reflection on individual behaviours; provide students with opportunities to develop their professional communication skills in giving and receiving feedback; and for feedback recipients to reflect on their peers’ comments and improve their teamwork behaviours [1, 2]. A well-developed process, with student engagement in the provision and receipt of peer feedback, is considered key to the success of TBL [2, 3]. Unlike traditional classes, where students are accountable only to the teacher, TBL requires students to also be accountable to their peers. The structured format of TBL, with three key phases: (1) preparatory, (2) readiness assurance and (3) application include elements that require students to work in teams, synthesise information, and communicate with each other. Varied opportunities exist in TBL for students to develop a range of professional skills relevant to future health professional practice: individual accountability, problem solving, communication, teamwork and organisational skills [4].

Giving and receiving peer feedback has the capacity to provide an effective learning experience for students, creating reflective learners, who analyse their own performance [5]. Additionally, the recipients’ perception of the quality of the feedback is important in prompting a positive view towards change [6]. Students’ perception of the quality of feedback provided by peers has been reported in several studies as more valuable and relevant than feedback provided by faculty [7]. However, there are some negative aspects reported regarding the process and student response to peer feedback, such as poorly conveyed feedback and apprehension of being criticised by peers [8–10]. Peer feedback may be lenient for a number of reasons, including social discomfort, insufficient preparation and training, and the associated responsibility [11–14]. Where there is a lack of honesty in peer feedback, performance and behaviours may remain unchanged [8].

Peer review in TBL involves each student formally grading the contribution of their team members. Literature reports varied approaches in the way in which the peer review score is generated. However, these methods are generally designed to measure students’ contributions to team cohesion and productivity, as perceived by their teammates, rather than student knowledge. For example, in “Michaelsen’s approach”, each team member is asked to assign a score to each of their team members [15]. Consequently, each team member’s peer evaluation score is the average of the points they received from the members of their team. This evaluation score typically composes a fixed portion of the students’ final mark (normally 5–10 %). Another method of peer review in TBL, the “Texas Tech method” includes a mix of qualitative and quantitative feedback [16]. Feedback is not assessed by an academic, and the peer review score contributes to the students’ final mark. The “Koles method” of peer review includes both quantitative and qualitative feedback [1, 16]. The quality of the feedback provided by peers is rated by the facilitator. Both this score from the facilitator, and the feedback score that is received from the peer, contribute to the final ‘peer review’ score. The benefit of this method is that the peer evaluation score depends on both the quality of the students’ performance as judged by their peers, and the quality of one’s own feedback. Hence, professional skills in both giving and receiving feedback are enhanced.

Despite the recommendation that a peer review process is key to the success of TBL, evidence suggests it remains a difficult task to incorporate [2]. In a 2014 systematic review on the essential elements of TBL, less than half of the 20 included articles reported using peer review process, which typically took place only on the last day of the course [2]. Although TBL has been implemented at Sydney Medical School since 2016, a peer review process was not implemented until 2018, with few published examples evidencing success.

Theories underpinning teaching and learning methods provide useful frameworks to analyse educational practices [17]. Biggs (2003) ‘3P model’ proposes that students’ characteristics, values and learning environment (presage) influence their approaches to learning (process), that in turn influences the achievement of learning outcomes (product) [18]. That is, students’ motivation and strategies employed in learning is dependent upon the integration of presage, process and product, facilitated by the design of the learning activity.

This study took place at Sydney Medical School, where TBL is used to teach integration of basic sciences and clinical concepts in the first two years of the medical program. In this context, we sought to explore a newly implemented process of peer review within TBL. The aim of this study was to assess students’ ability to provide qualitative written feedback to their peers in TBL, and to explore students’ perception of the process, their experience and outcomes, using the theoretical framework of Biggs ‘3P model’ [18]. Our specific research questions were:

1. What is the quality of students’ feedback in the TBL peer review exercise, and does students’ ability to provide feedback improve over time?

2. What are students’ perceptions of giving and receiving written feedback from their peers in the TBL peer review exercise?

## Methods

### Participants

In 2019, all Year 2 students (*n* = 255) were required to participate in the peer review exercise in TBL. This cohort of students had previously participated in TBL peer review exercises in Year 1 (2018) of the Sydney Medical Program (SMP).

#### Theoretical framework

The theoretical framework used in this paper is based on social constructivist learning theories, which emphasises the social nature of students’ experiences. How learners conceptualise the giving and receiving of feedback is shaped by their prior experiences, strategies for learning and motivations. They are also impacted by how they experience the teaching context including learning activities and assessment, and expectations on behaviours. This intersection of feedback between various prior and ongoing learner experiences resonates with Biggs 3P model of teaching and learning [18]. The student experience of feedback within the TBL per review process, is conceptualized by using an adapted 3P model comprising presage, process and product factors [18, 19]. Within our context of the TBL peer review process, we used this model to conceptualise the student experience of giving and receiving feedback [19]: In this framework:

- **Presage**: refers to students’ individual characteristics, such as existing knowledge, skills, values and approaches to learning. Presage is influenced by the students’ previous feedback experiences, capacity to engage with feedback, and their motivation for self-improvement in response to feedback. Other influences include the environmental factors, such as the teaching context, curricular and assessment features, and their alignment with student expectations.
- **Process**: refers to students’ experiences of learning activities, including their expectations, the perceived value of the task and motivation to participate in the task; their response to the feedback, and making sense of feedback.
- **Product**: refers to students’ attainment of the learning outcomes, the impact of the feedback on the individual learner in both the short and longer term. This is influenced by both the ‘presage’ and the ‘process’. How well the presage and process factors are managed by teachers and learners, profoundly affects the impact of feedback.

### Research Context

**Fig. 1:**
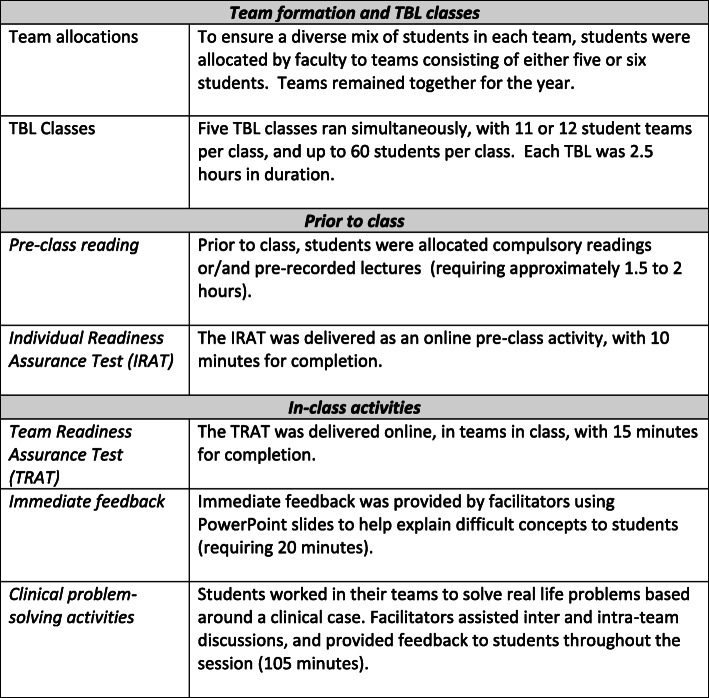
SMP TBL course design and process

The study took place in 2019, in Year 2 of the SMP, a four-year MD graduate entry program. TBL classes are attended by students approximately once per week throughout the academic year. Five TBL classes (2.5 h in duration) are held simultaneously, with approximately 60 students per class. Each classroom consists of 11 or 12 student teams, with five or six students per team. Student teams are allocated by faculty, and remain permanent for the academic year. Each TBL class has three facilitators (one medical consultant, one registrar and one basic scientist). The TBL process has been extensively reported previously [20] and is summarised in Fig. 1. In this context, the peer review exercise is a mandatory assessment within the Personal and Professional Development (PPD) theme of the medical program. It is designed to promote professional behaviours within the TBL and to enhance student ability to give and receive feedback.

### Peer review process

Twice yearly, Year 2 students were required to perform peer review using the online tool Sparkplus [21]. Peer review was undertaken at completion of the first two teaching blocks (Neurosciences and Endocrinology), which included 12 TBL sessions; and again, after completion of another two teaching blocks (Renal/Urology and Gastroenterology), which included eight TBL sessions. The students had already completed peer review twice in Year 1 (2018), and were familiar with the process. They had also received a briefing on the process, instructive lectures in Year 1 on giving and receiving feedback within the PPD teaching theme, and were given a further example of constructive feedback in the outline of this assessment task.

#### Peer review assessment task

**Fig. 2:**
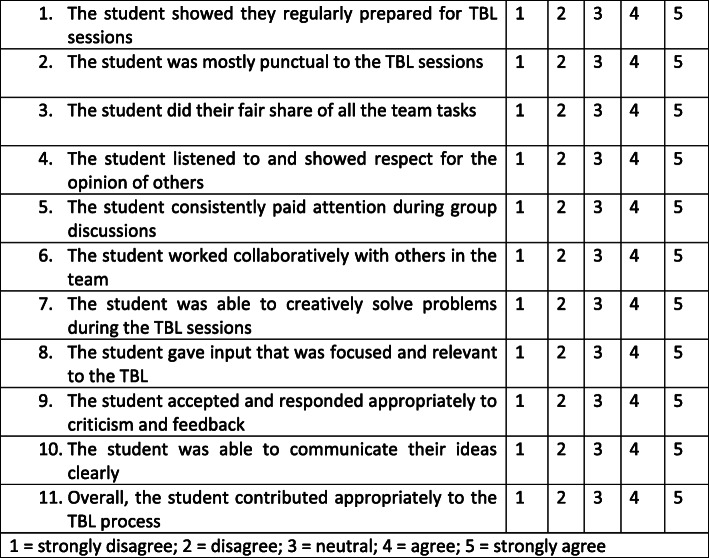
Questions and rating scale used in the validated TBL peer feedback task
- Provide constructive and professional written feedback on the contributions of at least two team members of their choice (limit of 200 words per student). The feedback was anonymous. It was requested that feedback provided by students should be descriptive, honest, and non-judgmental in nature. Examples of constructive and professional feedback were provided to students.

Students were required to provide and receive feedback on their professional learning behaviours within their team. Students were required to:

- Self-assess their own contributions to the team process.
- Rate all fellow team members on their contributions to the team process, by responding to 11 statements (Fig. 2), using a Likert scale of 1 to 5, with 1 being ‘strongly disagree, and 5 ‘strongly agree’.

Although the focus of this paper is on students’ qualitative responses, Fig. 2 demonstrates the scale for the peer assessment of professional learning behavior in a TBL group. The TBL professional behaviour scale was modified from previously validated scales for small group learning [19, 22] and contextualized to this TBL setting. A Delphi method, involving six senior academics was used to develop the domains and the items. The final version of the scale used in this study included 11 items across five domains: responsibility, respect, collaboration, critical analysis, and self-awareness through the TBL process.

### Study design

We undertook a mixed methods design. First, we evaluated the amount and quality of provision of feedback using a feedback evaluation instrument, which we validated as part of this study. Second, we undertook a qualitative analysis of focus group data using the modified 3P framework to seek explanations for the feedback behaviours, both in giving and receiving feedback.

### Feedback evaluation instrument development

An instrument to gauge the quality of the open comments from the peer review was developed by the first, second and third authors (AB, CR, SL), modifying the work of Guatheir et al. (2015) and Abraham & Singaram (2019), in light of our conceptual framework [23, 24]. We rated the feedback across four domains, assigning a mark from 0 (did not attempt) to 3 (student remarked specific examples):

1. *Behaviours – What was done well?*
  (A description of the behaviours around which feedback was given)
2. *Gap – What was not done well?*
  (The recognition of a difference between the behaviours displayed and that of a comparative standard)
3. *Action – What can be improved?*
  (Using the feedback to create a future expectations)
4. *Responsiveness – Was the feedback provided professional, with specific detail?*

Instances where feedback was thought to breach widely available medical school standards of professional behaviour were also recorded for each feedback instance. These breaches were categorised into three broad categories: feedback comments copied to provide feedback to multiple students (A); parts of comments copied to provide feedback to multiple students (B); and unprofessional comments (C), for example ‘brings nice cakes to TBL’, with no useful feedback. An ‘Other’ category was also made available for reviewers to record any instances of breaches of conduct outside these prescribed categories.

### Calibration of feedback rating

 Reviewers (AB, CR, SL) initially undertook a calibration exercise, whereby each reviewer independently evaluated the same 50 instances of student feedback and assigned a mark. Descriptive analyses of students’ feedback scores by the fifth author (TC) was provided to reviewers to alert reviewers to any discrepancies in their scoring as well as identify any hawkish or dovish marking tendencies. Results were shared with reviewers who discussed those marks where there was a discrepancy of more than 2 points. Following the calibration exercise, reviewers evaluated the quality of the student feedback provided from the first TBL peer review exercise (n = 695 comments) and the second peer review exercise (*n* = 827 comments).

### Focus groups

Three focus groups were held with 23/255 (9 %) participants. Of the 23 participants, 12 were male and 11 were female. The focus group questions were each one hour in duration, conducted by the first author (AB), using a semi-structured interview schedule, designed specifically for this study to explore students’ perceptions of the peer review process, and their experience of giving and receiving feedback from their peers. Data were transcribed verbatim. After immersing themselves in the data and reflecting on their own fields of practice, the first, second and third authors (AB, CR, SL) used framework analysis to code a portion of the dataset independently, using Biggs 3P model [18] as a theoretical framework to identify recurrent themes and subthemes, as a basis for interpretation [25]. Once meaning and any sources of divergence in the data had been negotiated between the researchers, the first author applied the coding framework to the whole dataset [25].

### Ethics approval

 Ethics approval was gained from the University of Sydney Human Research Ethics Committee.

## Results

### Quality of qualitative feedback within the peer review

In order to address our first research question, mean ratings of the quality of student peer feedback across each of the four assessment domains are presented in Table 1. This shows that as a cohort, students scored reasonably high in both the Behaviour and Responsiveness domains, but very low in both Gap and Action. In all domains, students were awarded greater marks for their feedback in the first peer review exercise compared with the second peer review exercise.

**Table. 1:** Mean student feedback score across four domains from the first peer review (*N* = 695) and second peer review (*N* = 827)

Domain	Session	Mean
Behaviour	1	1.92
	2	1.84
Gap	1	0.26
	2	0.15
Action	1	0.20
	2 (n = 826)	0.07
Responsiveness	1	1.73
	2	1.65

Scoring scale: a mark from 0 (did not attempt) to 3 (student remarked specific examples)

**Fig. 3:**
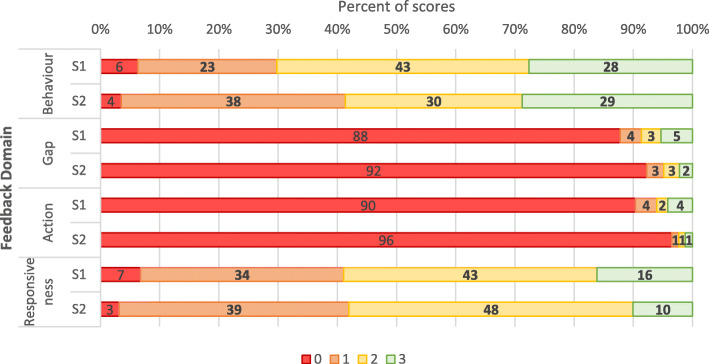
Peer Student Feedback score percent by domain for Session 1 S1 (*n* = 695) and Session 2 S2 (*n* = 827). Feedback was rated across the 4 domains, assigning a mark from 0 (did not attempt) to 3 (student remarked specific examples)

The low scores for both the Gap and Action domains in large part due to the overwhelming number of ‘0’ scores, indicated that students were not engaging with these aspects of feedback (see Fig. 3). This result identifies a need for a greater focus on facilitating constructive Gap and Action feedback among medical students.

**Fig. 4:**
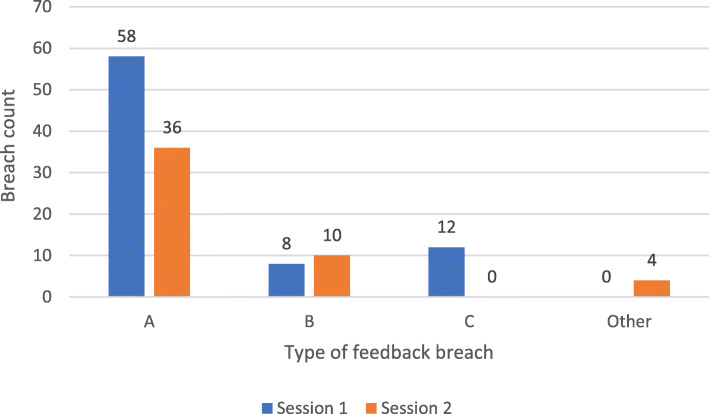
Comparison of instances of unprofessional feedback in Session 1 (*n* = 695) and Session 2 (*n* = 827), **A** represents comments copied across multiple feedback; **B** parts of comments copied across multiple feedback; C unprofessional comments; Other records of instances of breaches of conduct outside these prescribed categories

There were, however, fewer episodes of unprofessional feedback in session 2 than in session 1 despite nearly 200 additional feedback instances being marked in session 2 (Fig. 4).

In summary, our estimation of the quality of feedback in TBL suggests that students were comfortable identifying positive learning behaviours of their peers, but reluctant to identify any areas for improvement and even less to suggest ways in which that improvement might be made. A large proportion of students were not demonstrating responsiveness in providing enough specific detail in their feedback to be of value to the receiver. Students’ engagement with the task did not improve over the year. The most common breaches of professionalism in giving feedback was cutting and pasting the same, and often banal feedback (Fig. 4) to each peer in the TBL group.

### Students’ perception of giving and receiving written feedback in the TBL peer review exercise

In order to address our second research question, focus group findings are presented, illustrating the pertinent subthemes using the theoretical lens: ‘3Ps’: presage, process and product [18].

## Presage

### The task of providing feedback on their peers’ professional behaviour

Students felt it was more difficult to provide peer feedback on students’ behavior and professionalism, compared to feedback on their peers’ knowledge and skills.

*The TBL feedback you give is your punctuality, your contribution, your professionalism, and it’s a bit more personal rather than – I don’t mind someone saying, oh, you took a bad history - - - you could do this, this, and this to improve. But if someone said, I don’t like you in my group, you’re a dead weight….*

### Potential impact on peer’s academic record

Some students suggested that they were uncomfortable providing negative feedback to their peer in a written format, as they perceived it could impact on their academic record, or be taken out of context and have unintended consequences.

* To be honest, I felt like I just wouldn’t give harsh criticism because that would be really mean and really - if I had an issue with someone, honestly, with their work ethic in the group I would probably just say – what are you doing? I think people aren’t going to give too much constructive feedback in that kind of format because it’s written. And I personally wouldn’t be like, you don’t try in TBL. You should contribute more.…. I don’t want it really on their record, like I feel it’s not my place.*

### The influence of the large class TBL environment

 Students felt that some students’ behaviour was influenced by the large TBL classroom environment, compared to the clinical environment, where there are expectations around social behavior, appropriate to patient care.

*I do wonder if anything would change if you were in that smaller environment. I mean, again, no one slacks off in clinical because you’re all standing. You’re moving around in the wards. And you’re in a small environment in front of a patient. So, there’s sort of like there’s expected social behaviour that you uphold. But in a TBL room of 50 people it’s pretty easy to tune out the – you only often need about ten people speaking up in a TBL for it to breeze through two hours, if that.*

In summary, students strongly related the giving and receiving of feedback to their small group teaching in the clinical setting. Using this measure, students viewed provision of feedback on professional behaviours in a TBL group as an unclear concept, and appeared willing to tolerate a range of behaviours to avoid the perceived responsibility of written peer feedback.

## Feedback process

### The task of providing written feedback to peers

 While the students felt comfortable providing verbal feedback in clinical tutorials, they felt less comfortable providing written feedback in the TBL setting. This is likely in part due to the closer supervision in the clinical setting and increased accountability.

*Every time we take a history and then our tutor asks us individually, what did you think? And then asks the group for feedback. ….How would change their approach or how would you improve it? Maybe that’s something that could be implemented in TBL because, I think when you’ve got a face to face, like …. like in clinical…. you’re not painting everything positively. Like if I know something they can work on and something that they can….like I say, “You know, that was a great differential list, but I think …”.*

### Students didn’t feel that the feedback is read by academics to review its quality

Some students felt that monitoring by faculty, and appropriate follow up actions, should be part of the process. They felt that the peer feedback exercise was a low stakes assessment, where the quality of responses made little difference, and there was no likelihood of any consequences, even where professional misbehavior had been reported.

*Sometimes I also think it feels like that they’re not actually read… so it doesn’t really matter what’s written…. So, then it sort of feels like it’s just a tick box task, and no repercussions either. No one really wants to build in repercussions for not doing it properly, but maybe that encourages better engagement with the task.*

### Taking the task seriously

Students indicated that there was a more widespread lack of professional behaviour among students in provision of feedback.

*There’s some people that just kind of made a joke of it…. like there was no actual constructive criticism. And so then like, when you actually gave constructive criticism, like a couple of people in my group did, then the people who were joking around…. were a little bit offended I guess because they were like why are you saying this about me when I just like only like made jokes and said nice things about like everybody…. So, like, if everybody was like –actually gave constructive feedback then I think it would be better.*

In summary students’ experiences of the peer review learning activities were variable because of their expectations of appropriate behaviour in large class TBLs, and the perceived value of the feedback task to their own learning. There was little to suggest that students had responded to the feedback that they had been given other than to see it as an endorsement of their existing behaviours. While some students felt uncomfortable in an exchange of feedback with a peer where one had made a greater effort, there is little evidence of change in feedback behaviours.

## Product

### Impact of giving critical feedback on group dynamics

Students felt that if they provided critical feedback to their peers during the year, this may negatively impact the team dynamics. They expressed a sense of social discomfort at needing to again work in their teams after providing any negative feedback mid-year. They also felt that some individuals aren’t open to receiving constructive feedback.

*My main issue is ….comments released in the middle of the year,… if you gave legit feedback, like you don’t pay attention, you don’t contribute - if like anybody said anything remotely negative. Now your. team members know that, I just think that if you’re going to ask for like constructive, real feedback, it needs to be given at the end of the year. So it doesn’t have any…repercussions on like the group dynamics. Some people don’t take constructive feedback very well.*

### Students recognized the need to learn to respond appropriately to feedback

 Students recognised that learning how to receive feedback was an important part of their professional development and part of their future careers – to learn how to receive feedback from their colleagues, and also from patients.

*It’s just a little bit awkward. But the problem is with the people who can’t really – take the feedback, because it was all written really nicely, from like the people who did give the real feedback. It’s just like the people who received it I think weren’t expecting real feedback. No one’s talked about the elephant in the room. Far out I mean we’re going to be given feedback for the rest of our careers. Might as well start now….Patient feedback… feedback from your colleagues about how you’re doing. I don’t mind having feedback.*

Students also recognized that without feedback, negative behaviours may remain unchanged.

*…people can’t change behaviour (without feedback). So, if it’s constructive feedback…they have to actually learn to take it properly*.

### Assessment of feedback and addressing poor behaviours

Some students felt that there was a potential benefit of academic ‘policing’ of professional behaviours, which might reduce commonly observed unprofessional learning behaviours such as use of social media in class, impacting student contributions.

K*nowing that you’ll have the opportunity to comment on your peers’ performance and have faculty actually read it, I think might motivate some people. Like last year I had people sitting on their phones the entire time, or people who just wouldn’t contribute, or made our lives a bit more difficult….*

In summary, some students saw the peer feedback process as a low stakes assessment of professional behaviours. They had less insight into the value of achieving learning outcomes of giving and receiving feedback, and the impact of the feedback on the individual learner in both the short and longer term, and were skeptical of the impact on team dynamics. However, many students rationalised that it was not their responsibility to manage the feedback.

## Discussion

This study sought to explore medical students’ ability to provide qualitative feedback to their peers in TBL, and their perceptions of giving and receiving feedback in the peer review process. While students demonstrated a reasonable ability to provide positive feedback to their peers, they were less prepared to identify gaps and needs for improvement. Notably, students’ ability to provide constructive feedback did not improve from the first to second peer review exercise. Students’ reluctance to provide negative feedback to fellow team members regarding their professional behaviour in TBL was influenced by the perceived difficulty of the task; anticipated social discomfort; prior experience of the negative impact on team dynamics; and the sense of responsibility in providing written feedback that may impact a peer’s academic record. However, students conveyed an awareness of giving and receiving feedback as professional skills needed in their future medical careers; and indeed, a desire to improve the teamwork behaviours of their peers during TBL. Students felt the process of providing feedback would be improved by utilising verbal feedback in the small team setting, with adequate and transparent monitoring by academics to ensure student accountability. In the theoretical lens we applied in this study, we adapted Biggs 3Ps as a framework to both describe and provide explanation of students’ perception of their experience within the context of ‘presage’, ‘process’ and ‘product’; [18] and consider how the peer review activity might be redesigned to improve student learning outcomes. Using the theoretical framework of the modified 3P model allowed us to make novel contributions to this theory, and provide practical considerations for other educators adopting peer feedback in the TBL process.

### Presage

Presage refers to students’ individual knowledge, skills, values, and approaches to learning; prior experiences of giving and receiving feedback; individual expectations; and the environmental influences on student learning. Students found articulating their peers’ professional behaviour difficult, and felt more comfortable providing feedback on knowledge and skill-based performance in the clinical setting. Additionally, students were averse to providing critical written feedback to their peers because of possible future assessment ramifications. It is well documented that students have concerns about passing judgement on the performance of peers, with greater hesitation to accept responsibility when students know each other [7, 26], as is the case in the small teams of TBL. Farland & Beck (2019) described implementation of a successful longitudinal TBL peer review process designed to implement continuous development of teamwork skills, involving 261 pharmacy student teams [27]. They demonstrated a capacity to improve teamwork outcomes through targeted training and coaching [27]. It appears that our student training needs in provision of feedback, particularly in providing feedback on professional behaviours were underestimated. Evidence suggests that with adequate training, the practice of providing and receiving feedback provides an effective learning experience that encourages self-reflection [6, 7, 28, 29].

### Process

Process refers to the students’ experiences of the learning activity, including their expectations, the perceived value of the task, and their motivation to participate. It has been suggested that to be successful, the peer review process of TBL needs to form an integral part of curriculum design that is clearly linked to other course components [30]. There is a need to balance the requirement to develop students’ professional skills in giving and receiving feedback, and development of an effective process that promotes student ‘buy in’. Students indicated that provision of verbal feedback to all team members as a group discussion would be more beneficial than the current method of providing individual qualitative written feedback to only two team members. They also felt that students should be more accountable for the quality of the feedback they provide to their peers. They suggested the process should be more transparent, with feedback monitored to some extent by faculty, with repercussions for reported unprofessional behaviour. However, prior work suggests that peer assessment of professional learning behaviours may be unreliable, and therefore is not appropriate for a high stakes summative assessment [22]. An example of a more open peer review process is provided by Schug et al. (2018), where TBL was implemented across an entire Research Methods course [31]. Students completed peer review midterm and at the end of the course regarding team members’ performance. After aggregate mean scores for each team were graphed and distributed to students, teams met to identify actions to enhance their teamwork [31].

Notably, students’ ability to provide constructive feedback did not improve from the first to second peer review activity. This is contrary to a recent peer review TBL study by Fete and colleagues (2017), involving pharmacy students, where students’ ability to provide constructive feedback improved over time. Fete et al. (2017) found that the inclusion of authentic and actionable qualitative feedback, with input from faculty advisors provided greater differentiation between student performance, held students accountable for their own behaviour, and provided opportunities for improvement and personal development [32]. However, the focus remained on individual contributions to teams, and did not provide a structured approach for students to discuss their teamwork behaviours and attitudes with the entire team.

### Product

Product refers to students’ attainment of the learning outcomes of the peer review exercise, as influenced by both presage and process; the impact of the feedback on the individual learner in both the short and longer term, and how management of the peer review exercise by faculty and learners, can affect the quality and impact of feedback. The utility of peer review in TBL is evident only when students begin to monitor their own behaviours and make adjustments in anticipation of future review, creating influence over the learning environment. Students who are conscious that their peer review will affect their own grades are more accountable for participating in the learning process [33]. Although students felt that the receipt of negative feedback by some students impacted negatively on group dynamics, they also acknowledged the need to improve the professional behaviour of individuals in order to improve teamwork. Additionally, students identified the skills of giving and receiving feedback as essential to their future medical careers. Although peer review is a common requirement among junior medical staff, they are often ill prepared for this aspect of their career. It is widely acknowledged that there is a need to further develop these transferable skills during university education and training [34].

Bushe & Ratta (2017) reported on their peer review process, completed at the final TBL session, where students discussed and evaluated overall team effectiveness, rather than each member’s contribution to the team [35]. However, this method avoids the need to provide constructive, individual feedback. The finding of students’ discomfort in provision of constructive feedback is not unexpected, given the social dimensions and pressure that exist within student teams [6]. Social pressures within student teams can influence the honesty of feedback, with criticism viewed as socially uncomfortable to both give and receive [8, 9]. If feedback is perceived as judgemental, and not relayed appropriately, it may result in deterioration of performance; and impact negatively on peer interactions [8]. However, giving and receiving feedback provides an important educational tool in developing professional competencies, and the more acceptable and transparent the peer review process, the more effectively students will incorporate the feedback they receive to improve their behaviours [36].

### Study limitations

 To our knowledge this study is one of the first to analyse and interpret students’ perceptions of the giving and receiving of written feedback in the context of TBL using a theoretical lens. Three areas of uncertainty in our findings are that, first, some of the nuances of student perceptions of the behaviours we were investigating might be lost in a focus groups compared with individual interviews. Second, that some of our findings may be specific to the particular context of our medical program. Third, in extending theory on applying the Biggs 3P model to students perception of feedback, we found that in our analysis, we did not find the suggested linearity between each of the Ps; in practice we found considerable overlapping between each of the levels.^18^ Nevertheless, we believe our findings provide both theoretical and practical guidance for educators to reflect on the use of peer feedback approaches both within TBL and other small group learning settings.

## Conclusions

Within the TBL process, our data shows that students demonstrated a reasonable ability to provide positive feedback to their peers about their learning behaviours. They were less prepared to identify areas and propose actions for improvement. In order to meet peer review outcomes more effectively, these findings indicate a modified process is needed to increase students’ motivation to engage and acquire appropriate learning strategies, teamwork behaviours and professional skills in feedback. Provision of a detailed orientation that increases student accountability for giving and responding to peer feedback to meet expected standards of professional behaviour, is needed. Furthermore, adequate provision of training to help students understand the mechanics of the peer review tool and the process of feedback may enhance the educational and professional outcomes of the peer review exercise. Further research could explore a transparent process that incorporates verbal feedback and discussion in teams, with more proactive management of the process and outcomes by faculty to facilitate student ‘buy in’.

## Supplementary Information

**Additional file 1:**

## Data Availability

Datasets supporting the conclusions of this article are included within the article. Additional data at the level of individual students is not available as per confidentiality agreements approved by the Human Research Ethics Committee, University of Sydney.

## Abbreviations

TBL: Team-based learning
PBL: Problem based learning
IRAT: Individual Readiness Assurance Test
TRAT: Team Readiness Assurance Test
SMP: Sydney Medical Program
