# Supplementary material for: Peer review in team-based learning: influencing feedback literacy
Source: BMC Med Educ. 2021 Aug 12;21:426. doi: 10.1186/s12909-021-02821-6 (PMC8359024; doi:10.1186/s12909-021-02821-6)
Supplement: Supplementary file 1 — Additional file 1: [file 12909_2021_2821_MOESM1_ESM.docx]

**Semi-structured interview guide the TBL peer review**

How did you find the task of providing feedback to your peers?

How did you find commenting on their professional behaviour?

How did you feel about receiving feedback from your peers?

Do you have any comments to make on the process and the task as a requirement of the TBL?

Do you know how others in the class felt about the task?

Do you think that it helps to develop and improve professional beahviours?

Are there improvements that you would suggest to the task and the process?
